# The Initiation, but Not the Persistence, of Experimental Spondyloarthritis Is Dependent on Interleukin-23 Signaling

**DOI:** 10.3389/fimmu.2018.01550

**Published:** 2018-07-09

**Authors:** Melissa N. van Tok, Songqing Na, Christopher R. Lao, Marina Alvi, Desirée Pots, Marleen G. H. van de Sande, Joel D. Taurog, Jonathon D. Sedgwick, Dominique L. Baeten, Leonie M. van Duivenvoorde

**Affiliations:** ^1^Department of Clinical Immunology and Rheumatology, Amsterdam Rheumatology and Immunology Center, Academic Medical Center/University of Amsterdam, Amsterdam, Netherlands; ^2^Department of Experimental Immunology, Academic Medical Center/University of Amsterdam, Amsterdam, Netherlands; ^3^Eli Lilly and Co, San Diego, CA, United States; ^4^Rheumatic Diseases Division, Department of Internal Medicine, UT Southwestern Medical Center, Dallas, TX, United States; ^5^Cancer Immunology and Immune Modulation, Boehringer Ingelheim Pharmaceuticals Inc., Ridgefield, CT, United States

**Keywords:** spondyloarthritis, animal models, HLA-B27 tg rats, IL-23R, IL-23 signaling, IL-17A, experimental spondyloarthritis

## Abstract

IL-17A is a central driver of spondyloarthritis (SpA), its production was originally proposed to be IL-23 dependent. Emerging preclinical and clinical evidence suggests, however, that IL-17A and IL-23 have a partially overlapping but distinct biology. We aimed to assess the extent to which IL-17A-driven pathology is IL-23 dependent in experimental SpA. Experimental SpA was induced in HLA-B27/Huβ2m transgenic rats, followed by prophylactic or therapeutic treatment with an anti-IL23R antibody or vehicle control. Spondylitis and arthritis were scored clinically and hind limb swelling was measured. Draining lymph node cytokine expression levels were analyzed directly *ex vivo*, and IL-17A protein was measured upon restimulation with PMA/ionomycin. Prophylactic treatment with anti-IL23R completely protected against the development of both spondylitis and arthritis, while vehicle-treated controls did develop spondylitis and arthritis. In a therapeutic study, anti-IL23R treatment failed to reduce the incidence or decrease the severity of experimental SpA. Mechanistically, expression of downstream effector cytokines, including IL-17A and IL-22, was significantly suppressed in anti-IL23R versus vehicle-treated rats in the prophylactic experiments. Accordingly, the production of IL-17A upon restimulation was reduced. In contrast, there was no difference in IL-17A and IL-22 expression after therapeutic anti-IL23R treatment. Targeting the IL-23 axis during the initiation phase of experimental SpA—but not in established disease—inhibits IL-17A expression and suppresses disease, suggesting the existence of IL-23-independent IL-17A production. Whether IL-17A can be produced independent of IL-23 in human SpA remains to be established.

## Introduction

IL-17A is a central driver of axial and peripheral joint inflammation in human spondyloarthritis (SpA) (1–3). Although the biology of IL-17A is well understood (4), the mechanisms underlying the pathological production of, and/or sensitivity to, IL-17A in SpA remains largely undefined. IL-17A production was originally reported to be crucially dependent on IL-23 stimulation of lymphocytes (5). Accordingly, inhibition of IL-23 in mice caused a significant decrease in IL-17A levels (6) and IL-17A-mediated immunopathology such as experimental autoimmune encephalomyelitis (6) and spontaneous colitis in Winnie mice (7). In line with these experimental data, emerging clinical trial results in human psoriasis (PsO) indicate that not only IL-17A blockade (8, 9) but also blockade of the p40 (10) or the p19 (11–13) subunit of IL-23 is highly effective.

In contrast to PsO, however, IL-23 and IL-17A do not appear to act in a linear axis in gut inflammation. Whereas targeting the p40 (14, 15) as well as the p19 (16) subunit of IL-23 reduced clinical and histological inflammation in human Crohn’s disease, targeting IL-17A (17) or IL-17RA (18) was ineffective or even deleterious, respectively. Consistently, several experimental colitis models revealed that absence or inhibition of IL-23 but not so much IL-17A or IL-17RA reduced gut inflammation (7, 19–21). However, the effect of IL-23 inhibition might be contradictive as Thomas’s group published earlier that IL-23 blockade did not have an effect on ileitis development in the IL-23-dependent curdlan-induced SKG mouse model (22). Mechanistic studies addressing this dichotomy revealed a role for IL-23-independent IL-17A in the maintenance of epithelial integrity in the gut (23, 24).

The extent to which IL-17A production is dependent on IL-23 in SpA remains unknown. In line with IL-23R SNPs being associated with susceptibility to ankylosing spondylitis (AS) (25), IL-23 overexpression was reported to induce SpA-like disease, including enthesitis and new bone formation in mice (26). Of note, this phenotype could not be confirmed, with other groups demonstrating that IL-23 overexpression instead induces a severely destructive polyarthritis, resembling more a rheumatoid arthritis (RA) phenotype (27, 28). In the curdlan-induced inflammation in SKG mice, not only gut inflammation but also SpA was suppressed by IL-23 blockade (21). In human SpA, targeting p40 (29, 30) as well as p19 (31, 32) improves peripheral symptoms in psoriatic arthritis (PsA). For axial disease, a small open label trial suggested efficacy of p40 blockade in AS (33); however, more robust data with both anti-IL23p19 or anti-IL12p40 are not yet available.

Considering this emerging evidence that the respective biologies of IL-23 and IL-17 are not always linear but instead partially overlapping, the aim of the present study was to assess to what extend IL-23 is required to drive IL-17-dependent pathology in SpA. To examine this, we used the HLA-B27/Huβ2m transgenic rat as a model for HLA-B27-associated peripheral and axial joint pathology (34). We previously demonstrated that this model not only displays the inflammatory features of SpA but also the prototypical remodeling structural phenotype (35). Innate immune triggering with heat-inactivated *Mycobacterium tuberculosis* increased and synchronized disease incidence, which allowed for therapeutic intervention studies (36). Intervention with anti-IL-17A indicated that both prophylactic and therapeutic IL-17A blockade reduced inflammation and pathological new bone formation in this model (37, 38). Here, we assessed the impact of an anti-IL23R antibody on the incidence and severity of disease as well as on the expression of IL-17A and related cytokines in both prophylactic and therapeutic treatment experiments in the *M. tuberculosis*-induced disease in HLA-B27/Huβ2m transgenic rat (further referred to as HLA-B27 tg rats).

## Materials and Methods

### Antibody Discovery, Neutralizing Activity, and *In Vivo* Serum Exposure

A functional neutralizing rabbit/rat IgG2a chimeric antibody was generated by Eli Lilly and Co. (SA, USA). Rabbits were initially immunized with recombinant human IL-23 R-Fc fusion protein (Gly24—Asp353) (R&D systems cat #1400-IR-050). Single antigen-specific B cells were sorted and both heavy and light chain of IgG was cloned and expressed transiently in CHO cells as a full rabbit mAb. Supernatant of the transfected CHO cells was used for screening of IL-23R binding to BA/F3 cells overexpressing human IL-23R, by flow cytometry. To test the neutralizing activity of the IL-23R Ab for rat IL-23R *in vitro* and *in vivo*, a chimeric rabbit/rat IgG2A Ab was generated by fusing the antigen-binding region of the heavy and light chains from the original rabbit mAb to the rat IgG2a constant Fc domain. Freshly isolated rat splenocytes were used for a functional *in vitro* assay. Cells were preincubated with anti-IL-23R Ab (in fourfold dilutions) first followed by stimulation with either recombinant mouse IL-23 (made internally at Eli Lilly) or recombinant human IL-23 (eBioscience, cat #14-8239-63) (final concentration 100 ng/ml). Rat IL-17A was measured by ELISA in the supernatant (catalog #BMS635, eBioscience). The anti-IL23R antibody showed inhibition of IL-23 induced IL-17A production, with a half maximal inhibitory concentration (IC50) of 0.014 µg/ml (Figure 1A in Supplementary Material). After *in vivo* treatment in rats (twice weekly 15 mg/kg for 5 weeks), serum samples were evaluated for anti-IL-23R antibody levels. ELISA plates were coated with 2 µg/ml of human IL-23R (R&D, 1400-IR) overnight. After incubation with rat serum samples, anti-rat IgG-HRP (Biolegend, 405405) at 1:30,000 was used for detection with TMB as substrate. For *in vivo* antibody exposure, serum levels for IL-23R antibody from the dosed animals were measured by ELISA. The results indicate a sufficient amount of antibody present in serum of all treated rats. Anti-IL23R levels in the serum reached concentrations of at least 2,000× the IC50 (Figure 1B in Supplementary Material).

### Rats

The Tg(HLA-B*2705, B2M)21-3Reh and Tg(B2M)283-2Reh rat lines (34) on Lewis background were bred and housed (four per cage) in individually ventilated cages at the animal research institute AMC. F1(21-3 × 283-2) male and female rats were used for experiments. All animal experiments were performed in duplicate, data from *in vivo* studies as presented in this manuscript were pooled from two independent experiments. All animal experiments were approved by the AMC Animal Care and Use Committee.

### Orchiectomy and Immunization

To prevent epididymo-orchitis development in the male rats (39), orchiectomy was performed using standard methods (Protocol obtained from Envigo, Horst, The Netherlands). Six-week-old rats were immunized with heat-inactivated *M. tuberculosis* (Difco, Detroit, MI, USA) in 100 µl Incomplete Freund’s Adjuvant (Chondrex, Redmond, WA, USA) *via* subcutaneous injection in the tail base as previously described (36).

### Treatment With Anti-IL23R

Rats were treated once weekly by intraperitoneal injection with 15 mg/kg mouse anti-rabbit/rat chimeric IL-23R or PBS as a vehicle control. Prophylactic treatment (*n* = 12 per group) started 1 week after immunization. Therapeutic treatment (*n* = 10 versus *n* = 7) started 1 week after 50% of arthritis incidence. Randomization was performed at the start of therapeutic treatment, by an independent researcher (Leonie M. van Duivenvoorde), based on arthritis severity scores (rats without arthritis were excluded from further analysis, with exception of the disease incidence analysis). In all experiments, treatment continued for 5 weeks. The prophylactic and the therapeutic intervention studies were both performed in duplicate: data from both experiments were pooled for analysis (indicated numbers per group are the total numbers of animals after pooling the data).

### Clinical Scoring

The presence of arthritis in the paws was determined clinically and digital swelling was measured with plethysmometry. Arthritis severity in each paw was graded 0–3 as described before (36). Cumulative clinical scores were calculated for severity analysis. Swelling in cubic centimeter was normalized either to the days before disease onset in case of prophylactic treatment or to the day of start treatment in case of therapeutic treatment. Spondylitis was determined clinically by swelling and bumps in the tail and scored yes/no. In case of humane endpoints, due to ethical considerations, rats were sacrificed with the last observation carried forward. Humane endpoints were defined as 15% bodyweight loss or two completely swollen paws for prophylactic treatment; and 20% bodyweight loss or two completely swollen front paws for therapeutic treatment. Clinical scoring was performed by one observer (Melissa Van Tok), blinded for treatment.

### Draining Lymph Node Analyses

Popliteal lymph nodes were collected for RNA isolation. RNA was isolated using TRIzol and an automated homogenizer followed by RNeasy micro column isolation (Qiagen kit). Gene expression was measured in duplex reactions using SYBR green primers for *il17a, il22, il17f, tnf, il6, ifng* with *gapdh* as a housekeeping gene (primer sequences are available upon request). The relative expression was calculated with the “2^−ddCt^ method” (40). From the repetitive experiments (one prophylactic and one therapeutic experiment), popliteal lymph nodes cells were also used directly *ex vivo* for qPCR array analysis (Rat Th17 gene array from Qiagen) according to the manufacturer’s instruction. Popliteal lymph node cells were also restimulated with 10 ng/ml PMA and 1 µg/ml Ionomycin for 48 h. Restimulated cells were stained for IL-17A (or IgG2a in a control panel) and analyzed on a FACS Canto II (for this purpose, Brefeldin A was added for the final 4 h of stimulation). Supernatants from similar cultures were used for luminex cytokine assay using Milliplex MAP Rat Cytokine/Chemokine Mag Bead Panel (BioRad, Cat No. RECYTMAG-65K), according to manufacturer’s instruction.

### Histology

Hind paws and tails were fixed in 10% formalin, decalcified in Osteosoft (Merck), and embedded in paraffin. Five micrometer sections were stained for hematoxylin and eosin or safranin O/fast green and semiquantitatively analyzed by two independent observers, blinded for treatment group, as previously described (35).

### Statistics

Data Were Analyzed Using GraphPad Prism 7 Software. Spondylitis and arthritis incidence were analyzed using a survival curve. Comparison of survival curves was analyzed using the Log-Rank (Mantel–Cox) test. Arthritis severity (arthritis score and hind paw swelling) was analyzed using the area under the curve followed by a Mann–Whitney *U* test. All other data were analyzed using a Mann–Whitney *U* test.

## Results

### Anti-IL23R Completely Prevents Spondylitis and Arthritis Development in HLA-B27tg Rats

**Figure 1:**
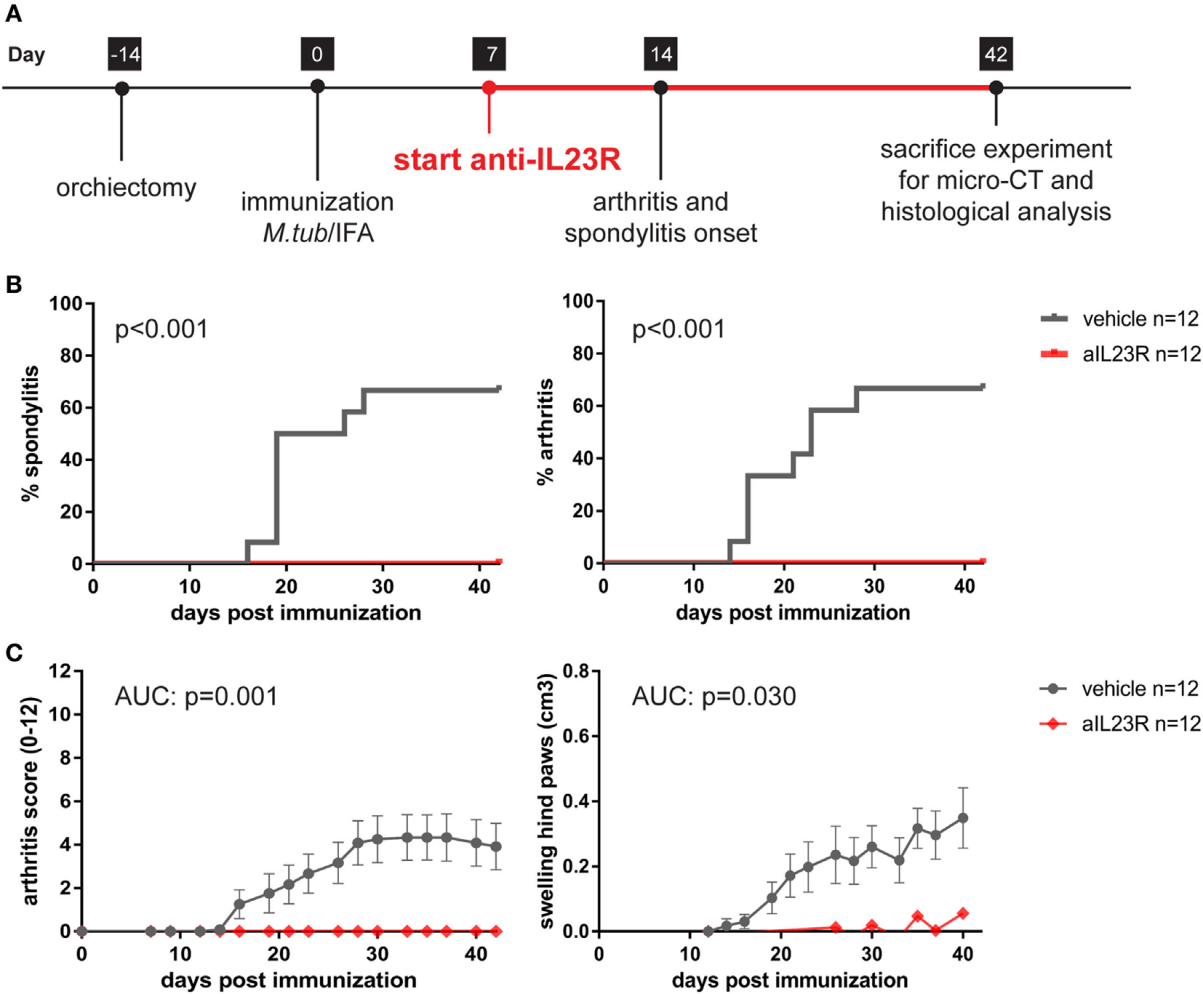
Prophylactic treatment with anti-IL23R prevents the development of spondylitis and arthritis. **(A)** Timeline of the experiment where HLA-B27 tg rats were immunized and prophylactically treated with anti-IL23R or vehicle for 5 weeks (*n* = 12 per group). **(B)** Spondylitis and arthritis incidence (survival analysis). **(C)** Arthritis severity as measured by arthritis score and swelling of the hind paws (data are mean ± SEM).

To assess whether IL-23 is essential for the development of spondylitis and arthritis, we prophylactically treated HLA-B27 tg rats with anti-IL23R. The treatment started after the immunization but before the onset of clinical symptoms (Figure 1A). In the vehicle treated group, 58 and 67% of the rats developed spondylitis and arthritis, respectively (Figure 1B). Arthritis severity, in terms of arthritis score and hind paw swelling, increased over time in the vehicle-treated group only (Figure 1C). In contrast, treatment with anti-IL23R completely protected against the development of both spondylitis and arthritis (Figures 1B,C).

### Therapeutic Blockade of IL-23R in the HLA-B27 tg Rats Fails to Suppress Arthritis and Spondylitis

**Figure 2:**
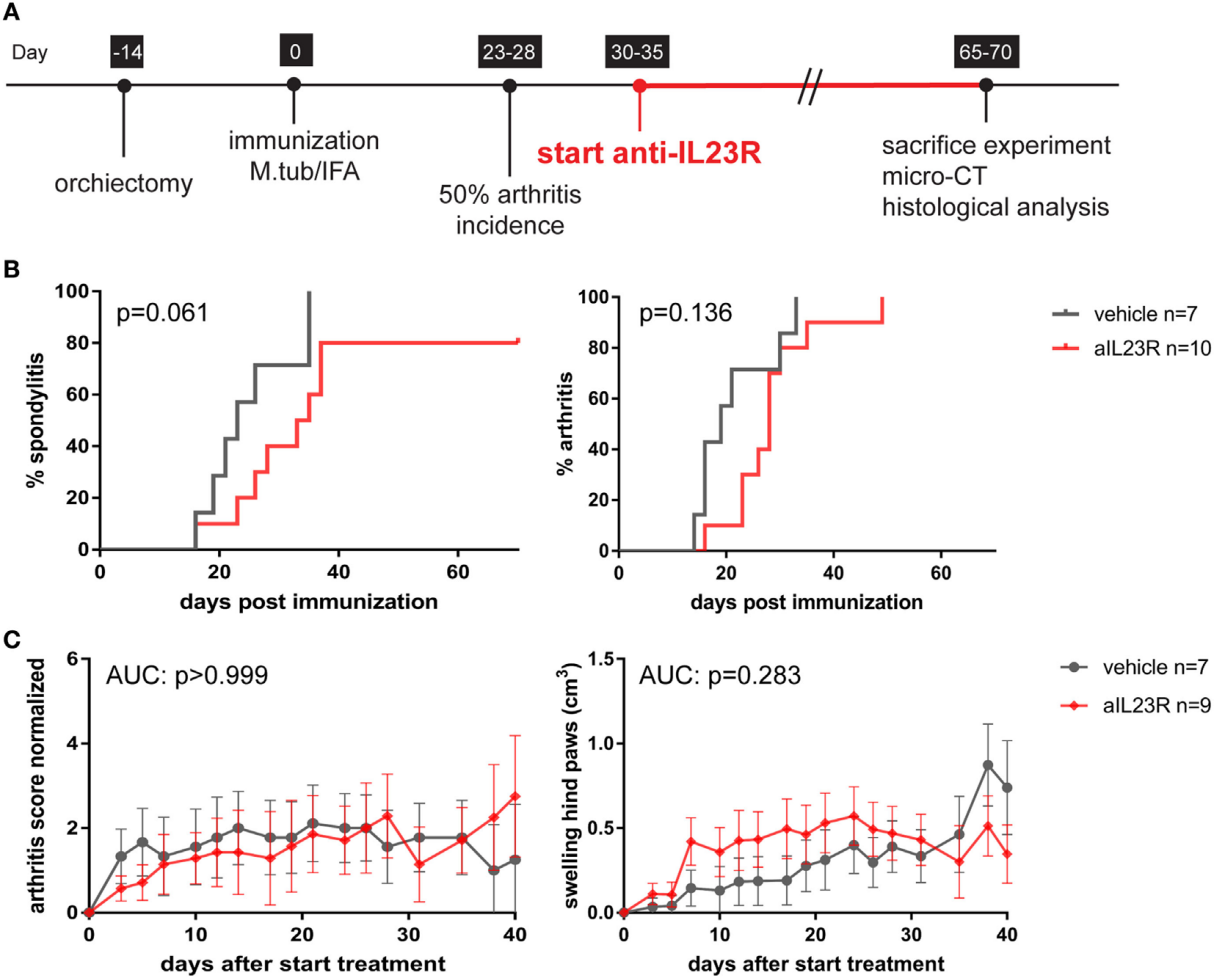
Therapeutic treatment with anti-IL23R does not affect established spondylitis and arthritis. **(A)** Timeline of the experiment where HLA-B27 tg rats were immunized and therapeutically treated with anti-IL23R or vehicle for 5 weeks (*n* = 10 and *n* = 7 per group, respectively). **(B)** Spondylitis and arthritis incidence (survival analysis). **(C)** Arthritis severity as measured by arthritis score and swelling of the hind paws (data are mean ± SEM).

**Figure 3:**
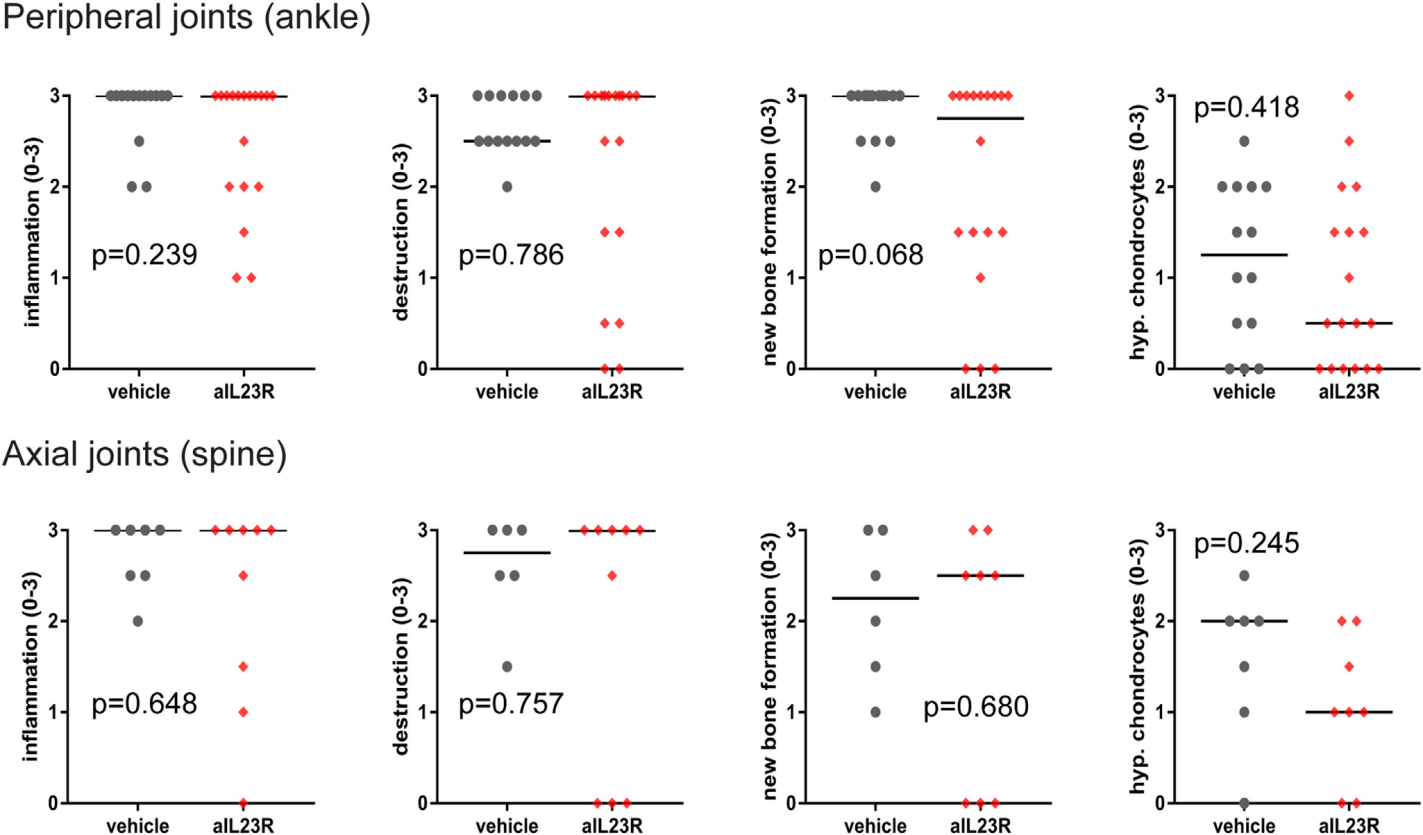
Histological analysis of peripheral and axial joints after therapeutic anti-IL23R treatment. Ankle and spinal sections from HLA-B27 tg rats were histologically stained and semiquantitatively examined for inflammation, destruction, new bone formation, and ectopic foci of hypertrophic chondrocytes (individual data points for each ankle joint or spinal sample and median are depicted).

To assess the role of IL-23 during disease progression, we next compared anti-IL23R to vehicle treatment in a therapeutic approach, with initiation of treatment after the onset of clinical symptoms (Figure 2A). Of the vehicle-treated rats, 100% developed spondylitis and arthritis. Similarly, the anti-IL23R-treated group reached an incidence of 80–100% for both spondylitis and arthritis (Figure 2B), with similar arthritis severity between the groups (Figure 2C). The absence of disease suppression by anti-IL23R in this therapeutic setting was confirmed by histological analysis of peripheral joints and spine at the end of the experiments: there was no difference between anti-IL23R and vehicle-treated animals with regard to inflammatory infiltration, bone destruction, new bone formation, and presence of ectopic foci of hypertrophic chondrocytes (Figure 3; Figure S2 in Supplementary Material). Analysis of serum anti-IL23R antibody levels confirmed similar exposure in the prophylactic and therapeutic study (Figure S1B in Supplementary Material). Moreover, measuring IL-23R and Rorc gene expression systemically, in draining lymph nodes, before disease onset (after immunization), at the end of the preventive experiment and after the therapeutic experiment revealed only an increase in IL-23R expression shortly after immunization (Figure S3 in Supplementary Material).

### Anti-IL23R Treatment Suppresses Downstream Effector Cytokines in a Prophylactic, but Not in a Therapeutic, Setting

**Figure 4:**
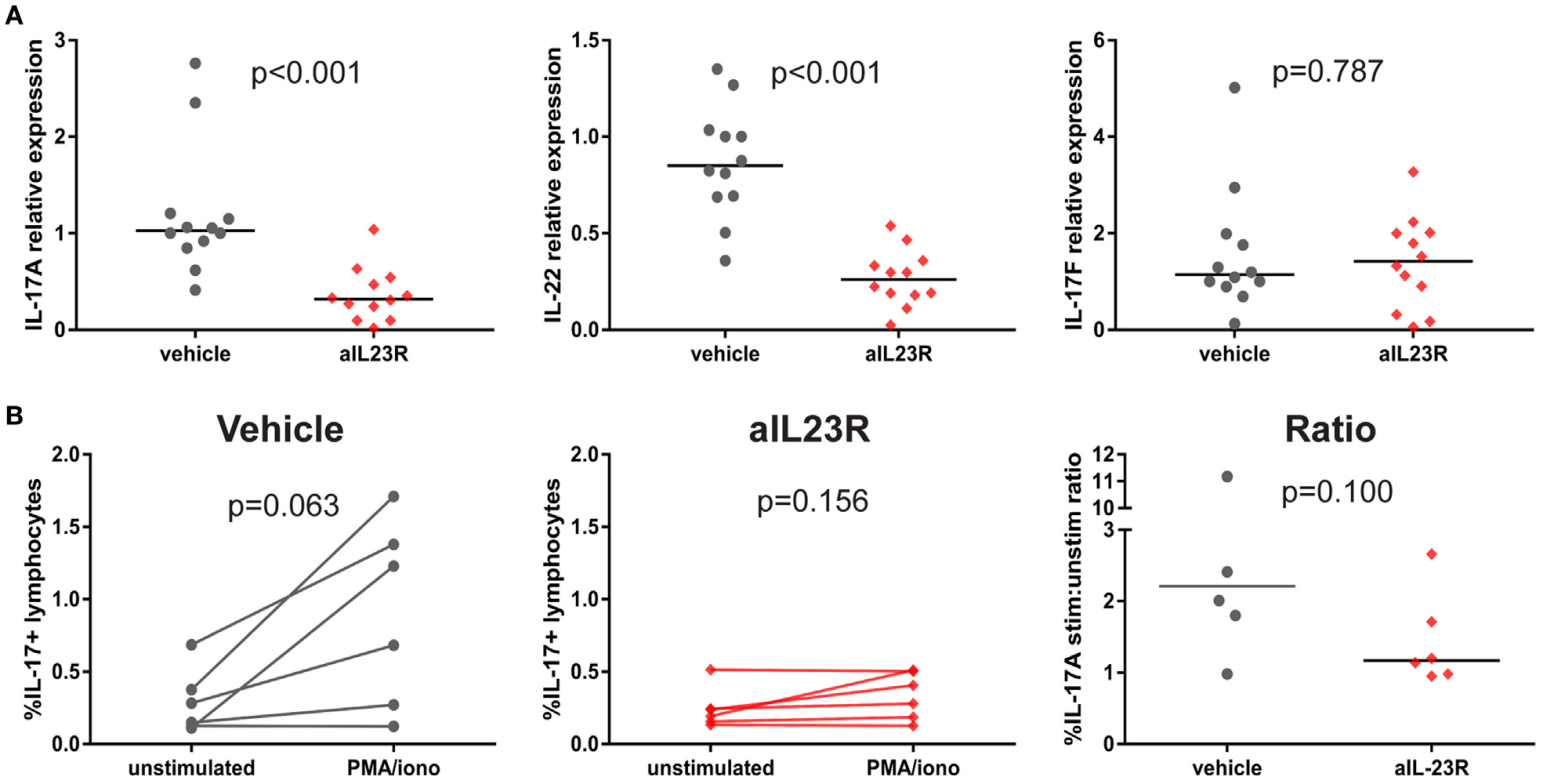
Prophylactic treatment with anti-IL23R, effect on downstream molecules in draining lymph nodes. **(A)** IL-17A, IL-22, and IL-17F gene expression directly *ex vivo* **(B)** IL-17A protein in draining lymph nodes in unstimulated (medium only) versus stimulated (PMA/ionomycin) condition in vehicles and aIL23R-treated rats and the ratio between the two groups (individual data points and median are depicted).

To assess whether blockade of IL-23R affected the downstream cytokines, including IL-17A, gene and protein expression analysis was performed on draining lymph node cells. In the prophylactic experiments, expression of *il17a* and *il22* mRNA was significantly lower in anti-IL23R versus vehicle-treated rats (Figure 4A). A similar trend was observed in splenocytes (Figure S4 in Supplementary Material). The expression of *il17f* (Figure 4A) and other pro-inflammatory cytokines such as *tnf, il6*, and *ifng*, was not affected by treatment (Figures 5B,C in Supplementary Material). To confirm the impact on downstream cytokines, lymph node cells were restimulated with PMA/ionomycin and analyzed for IL-17A protein production. In vehicle-treated rats, stimulation increased the percentages of IL-17A positive lymphocytes, while rats that were treated with anti-IL23R were less sensitive to this stimulation (Figure 4B). Together, these data indicate that prophylactic treatment with anti-IL23R suppresses the downstream cytokines IL-17A and IL-22.

**Figure 5:**
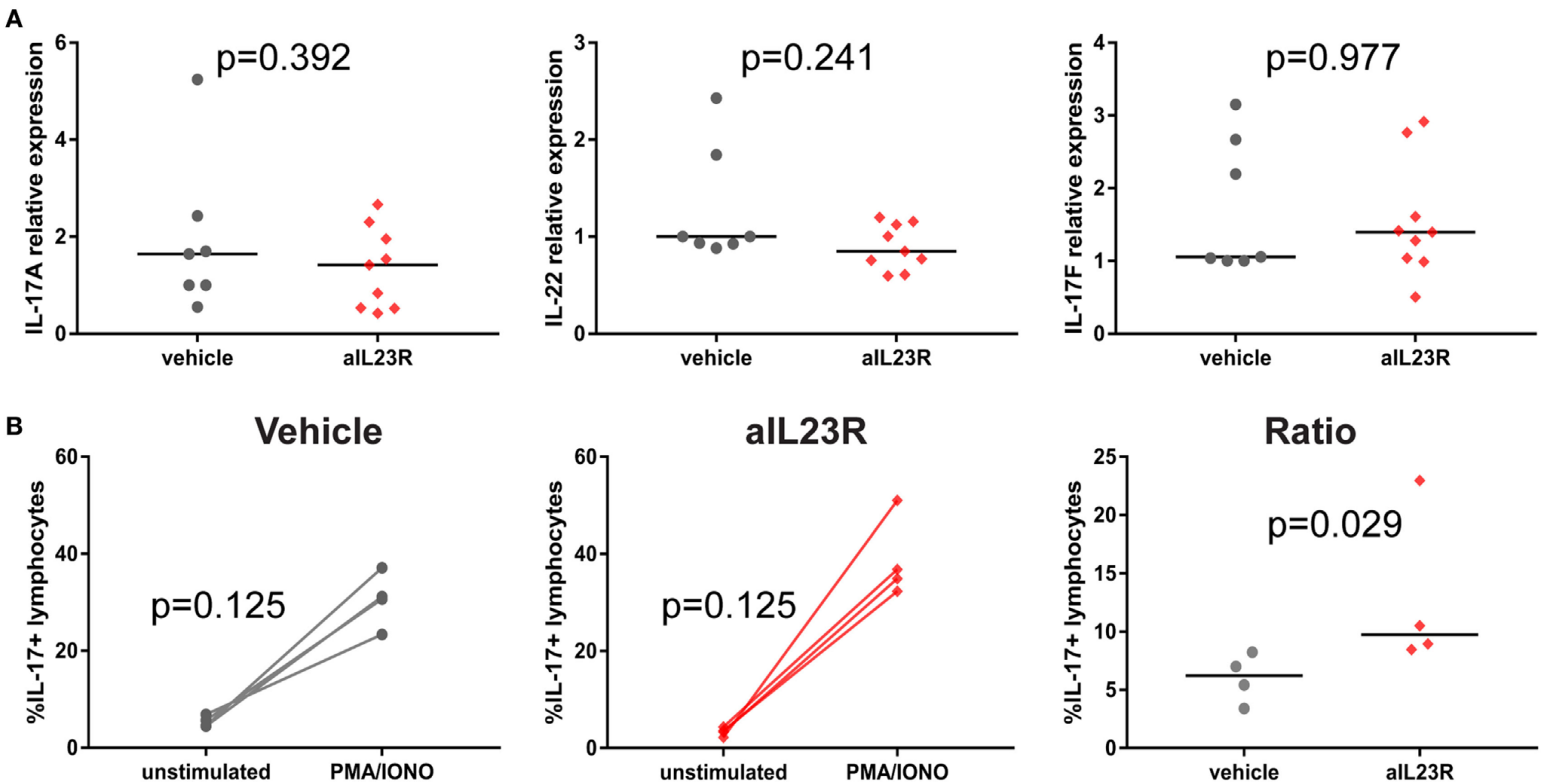
Therapeutic treatment with anti-IL23R, effect on downstream molecules in draining lymph nodes. **(A)** IL-17A, IL-22, and IL-17F gene expression directly *ex vivo*, **(B)** IL-17A protein in draining lymph nodes in unstimulated (medium only) versus stimulated (PMA/ionomycin) condition in vehicles and aIL23R treated rats and the ratio between the two groups (individual data points and median are depicted).

In contrast to the prophylactic experiments, a similar analysis in the therapeutic experiments showed that expression levels for *il17a, il22*, and *il17f* were similar in anti-IL23R and vehicle-treated rats (Figure 5A). Confirming these data, the percentage of lymphocytes producing IL-17A upon *ex vivo* PMA/ionomycin restimulation was not affected by anti-IL-23R treatment; on the contrary, the induction of IL-17A production was even more robust in anti-IL23R than in vehicle-treated animals (Figure 5B). Analysis of cytokine secretion in the supernatant confirmed similar IL-17A protein production in both groups, with similar findings for other inflammatory mediators including TNF, IL-6, GM-CSF, IFN-γ, and IL-10 (Figure S6 in Supplementary Material). Together, these data support the interpretation that, while preventive targeting of IL-23R significantly suppressed IL-17A and IL-22, therapeutic targeting did not affect these and other pro-inflammatory cytokines.

### Targeting the IL-23 Axis Increases IL-9 Without Affecting Th1 or Th2 Profiles

**Figure 6:**
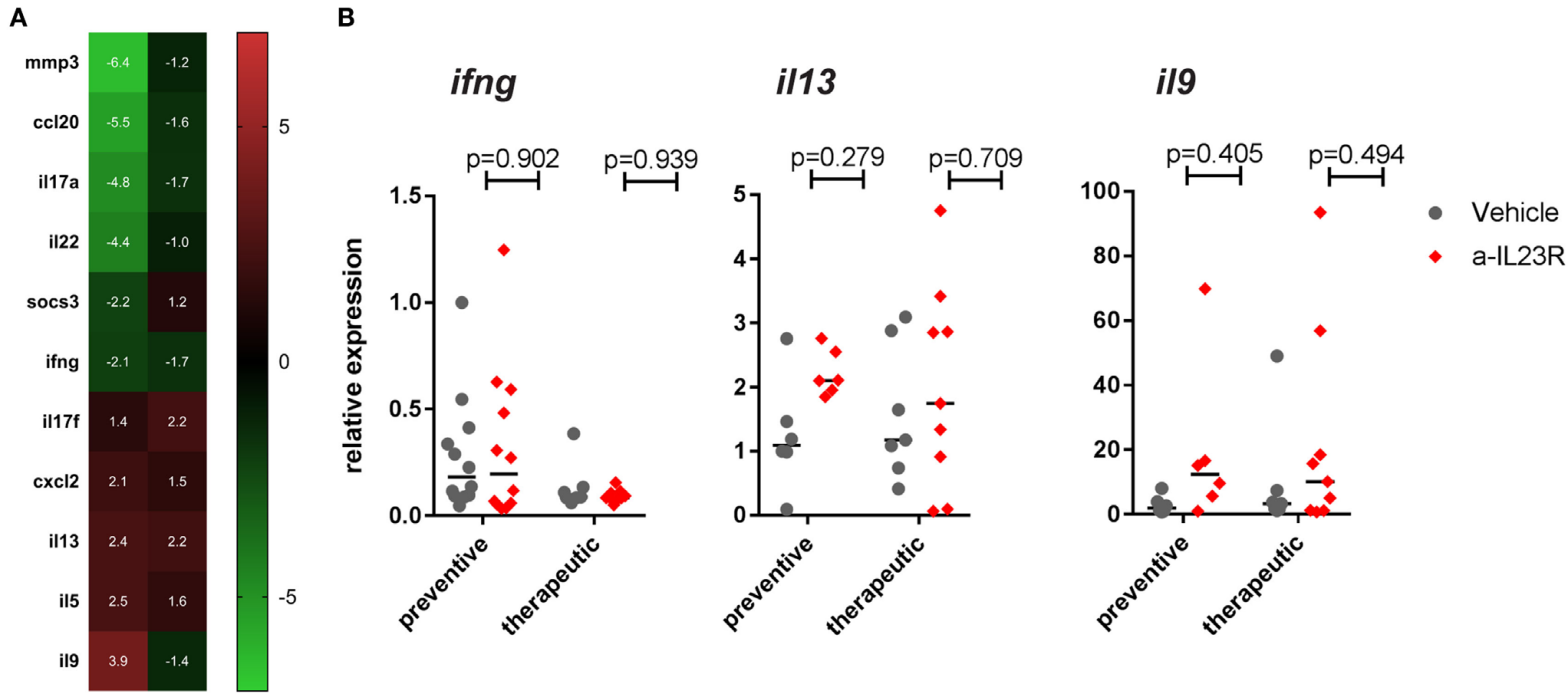
Th17 gene regulation in popliteal LN from prophylactically versus therapeutically treated rats. **(A)** Top genes (>2-fold up- or downregulated) in aIL23R treated versus control rats (data are fold change aIL23R versus vehicle). **(B)** Confirmation by qPCR on all samples for Th1/Th2 cytokines IFN-γ, IL-13, and IL-9 (individual data points and median are depicted).

To assess whether the differential impact of IL-23R blockade in a prophylactic versus therapeutic setting was restricted to Th17 cells, we performed a broad gene expression analysis to assess other T cell profiles. In line with the previous data, *il17a* and *il22* were suppressed in the prophylactic experiment, as well as the inflammatory markers *mmp3* and *ccl20* (Figure 6A). None of these changes were seen in the therapeutic setting (Figure 6A). The array data, together with confirmation by qPCR, showed minimal to no difference in either the prototypical Th1 cytokine *ifng* or the prototypical Th2 cytokine *il13* (Figures 6A,B). Moreover, the expression of the related transcription factors (*tbx21* and *gata3*) was also not affected (Figure S7 in Supplementary Material). The Th9 cytokine *il9* was strongly increased in the prophylactic experiment (3.9-fold) but not in the therapeutic experiment in the qPCR array analysis (Figure 6A); qPCR did not confirm the upregulation of *il9* by anti-IL23R (Figure 6B). Collectively, these data indicated that, although *il9* is upregulated, and there is no significant difference in Th1/Th2/Th9 cytokines between the prophylactic and the therapeutic study.

## Discussion

We have previously shown that treatment with anti-IL17A in both emerging and established disease resulted in decreased spondylitis and arthritis in the HLA-B27 tg rat (37, 38). In the present study, we asked to what extent is IL-23 required to drive IL-17A-dependent pathology in this model? We showed that IL-23R is crucial for the initiation of SpA, but not for the persistence of the disease. Interestingly, downstream effector cytokines, including IL-17A and IL-22, were downregulated only after prophylactic blockade of IL-23R—not after therapeutic blockade—despite levels of anti-IL23R antibody in the serum sufficient to block an IL-23 response. Consistent with our results, others showed that prophylactic IL-23p19 inhibition reduced clinical severity in the curdlan-induced SpA mouse model (21). Moreover, overexpression of IL-23p19 resulted in the spontaneous development of SpA-like symptoms (26). These data confirm that disease initiation is crucially dependent on IL-23. In contrast to the HLA-B27 tg model, therapeutic treatment with anti-IL-23p19 in curdlan-induced SpA in SKG mice induced a significant albeit small reduction in spondylitis and arthritis scores (21). However, the curdlan-induced SpA model arises through stimulation of dectin-1 (41), suggesting that this disease model is dependent on the IL-23 axis (42, 43), while disease pathology in the rat model studied here is dependent on the major susceptibility gene for SpA, HLA-B27. The difference in disease induction in these models potentially explains the difference in therapeutic treatment outcome. Others showed in mouse collagen-induced arthritis, the prototypical RA model, that prophylactic blockade of IL-23p19 was effective, while therapeutic treatment did not affect the disease course (44). However, multiple clinical trials (45, 46) have indicated the lack of efficacy in blocking IL-23/IL-17 signaling in RA. We show here, in a prototypical SpA model, that blockade of IL-23R is not effective in a therapeutic setting, while IL-17A blockade has been proven successful in this model (37, 38), as in human SpA (1–3).

Since we have previously observed that established disease in HLA-B27 tg rats is dependent on IL-17A (37, 38), it would follow that the lack of therapeutic effect in the present study is due to the persistence of IL-17A despite IL-23R blockade. Consistent with this interpretation, the transcription of IL-17A mRNA as well as translation of IL-17A protein after restimulation directly *ex vivo* indicated that IL-17A was still actively produced in the absence of available IL-23R. Moreover, we did not observe major changes in other pro-inflammatory cytokines that could account for the ongoing inflammation. Others have shown in an inflammatory bowel disease mouse model that γδ T cells were able to produce IL-17 independently of IL-23 signaling, which was shown to have protective effects by promoting the epithelial barrier function (23). Moreover, it has been suggested that prostaglandin E2 (PGE2) stimulation, independently of IL-23, can trigger IL-17A production in RA synovial fibroblasts (47), but several studies in mice indicated that PGE2 does need IL-23 signaling for stimulation of IL-17A production (48–50). Which of these mechanisms may be relevant to IL-23-independent IL-17A production in the HLA-B27 tg rat model of SpA is currently under investigation.

This study shows that IL-23R blockade is only sufficient to use in preclinical disease and not in full blown disease, as IL-17A is still produced in the therapeutic setting and disease activity is not reduced. This observation might be relevant to human SpA. While blockade of IL-17A is proven to be efficacious both in AS and PsA (1–3), data on the upstream blockade of IL-23 are only available for peripheral arthritis in PsA. Phase III trials with ustekinumab, an anti-IL-12/23p40 antibody, showed significant improvement of symptoms in active PsA (29, 30). Similarly, phase II trials with two different anti-IL-23p19 antibodies, guselkumab, and risankizumab, showed improvement of signs and symptoms of active PsA (31, 32). Patients are currently being recruited for two phase III trials with guselkumab in PsA (ClinicalTrials.gov identifier: NCT03158285 and NCT03162796). With regard to axial disease, a small open-label study with ustekinumab in AS suggested a beneficial effect (33); however, two large phase III trials with ustekinumab in axial SpA patients were terminated, apparently for lack of efficacy (ClinicalTrials.gov Identifier: NCT02407223 and NCT02438787). Similarly, a phase II trial with risankizumab in AS was completed in 2015 (ClinicalTrials.gov Identifier: NCT02047110), but data have not been released yet. It thus remains questionable whether IL-23 inhibition is effective in human axial SpA.

Although the effect of a-IL23R treatment in the prophylactic setting is obvious concerning clinical manifestations and histological analyses; interestingly, we were not able to observe a clear effect on gene expression levels of downstream targets, like Rorc, IL-17A, and IL-22 (Figure S3 in Supplementary Material). This might be due to several reasons, one obvious reasons is that total LNs or spleens were used for analyses, whereas only a very small portion of these cells are reacting towards IL-23R signaling. Another reason for the lack of an effect measurable by qPCR is that we did not analyze the appropriate tissues. All tissues used for these analyses were lymphoid tissues (popliteal LNs as well as spleen) and not targeted tissues, like the affected joints. For future experiments, we should measure either mRNA levels and protein levels in the targeted tissue as well. Nevertheless, we were able to show a decrease in IL-17A and IL-22 cytokine production after *in vitro* restimulation with PMA/ionomycin (Figure 4).

In conclusion, the data obtained in the HLA-B27 tg rat model of SpA confirms the concept of a partially overlapping but distinct pathobiologies of IL-17A and IL-23 and suggests that IL-17 inhibition may be more effective than IL-23 targeting in established SpA. The mechanism of IL-23-independent IL-17 production and the relevance of these experimental findings for human SpA need to be further elucidated.

## Ethics Statement

This study was carried out in accordance with the FELASA guidelines and recommendations. The protocol was approved by the AMC Animal Care and Use Committee.

## Author Contributions

MT and LD contributed to study design, data collection, analysis and interpretation, and were involved in writing of the manuscript. CL and MA contributed to the generation of the antibody, data collection, and analysis. SN, JT, and DB contributed to study design, data interpretation, and were involved in writing of the manuscript. DP contributed to data collection and analysis. MS and JS contributed to study design and data interpretation. All authors revised the manuscript, read and approved the submitted version.

## Conflict of Interest Statement

JT has license agreements with AbbVie, Anges, Inc., Celgene, and Novartis. SN, CL, and MA are employees of Eli Lilly and Co. MS received consultancy fees from AbbVie and Novartis. JS was a former employee of Eli Lilly and Company and continues to hold stock in the Company. DB is a part-time employee of UCB and received consultancy fees/grants from AbbVie, Pfizer, MSD, Roche, BMS, Novartis, Eli Lilly, Janssen, Glenmark, Boehringer-Ingelheim. MT, DP, and LD have no declarations of interest.
