# Supplementary material for: The Initiation, but Not the Persistence, of Experimental Spondyloarthritis Is Dependent on Interleukin-23 Signaling
Source: Front Immunol. 2018 Jul 9;9:1550. doi: 10.3389/fimmu.2018.01550 (PMC6046377; doi:10.3389/fimmu.2018.01550)
Supplement: Supplementary file 1 [file image_1.pdf]

A

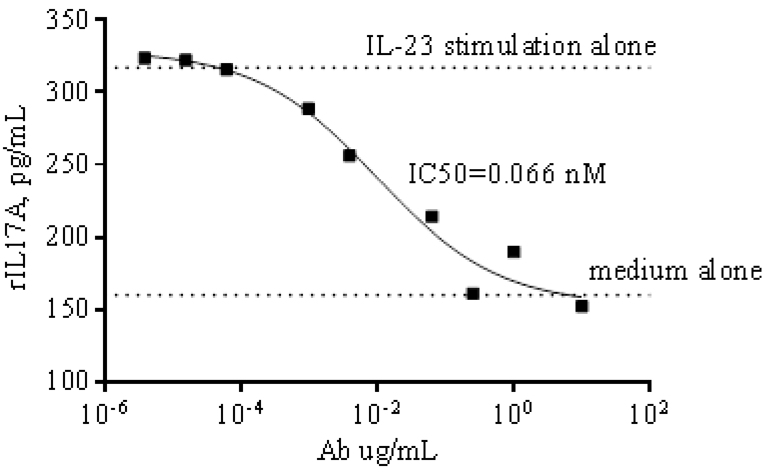

B

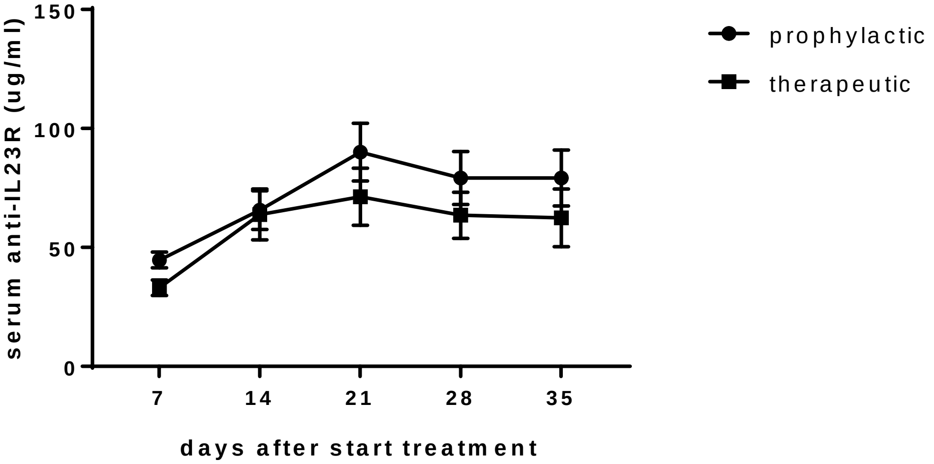

Suppl. Fig. 1 Neutralization capacity and serum exposure of the anti-IL-23R antibody. A. The anti-IL23R antibody showed inhibition of IL-23 induced IL-17A production, with a half maximal inhibitory concentration (IC50) of 0.014 µg/ml. **B.** After in vivo treatment in rats (twice weekly 15 mg/kg for five weeks) serum samples were evaluated for anti-IL-23R antibody levels. The results indicate a sufficient amount of antibody present in serum of all treated rats. Anti-IL23R levels in the serum reached concentrations of at least 2000x the IC50.
