# Supplementary material for: The Initiation, but Not the Persistence, of Experimental Spondyloarthritis Is Dependent on Interleukin-23 Signaling
Source: Front Immunol. 2018 Jul 9;9:1550. doi: 10.3389/fimmu.2018.01550 (PMC6046377; doi:10.3389/fimmu.2018.01550)
Supplement: Supplementary file 2 [file image_2.pdf]

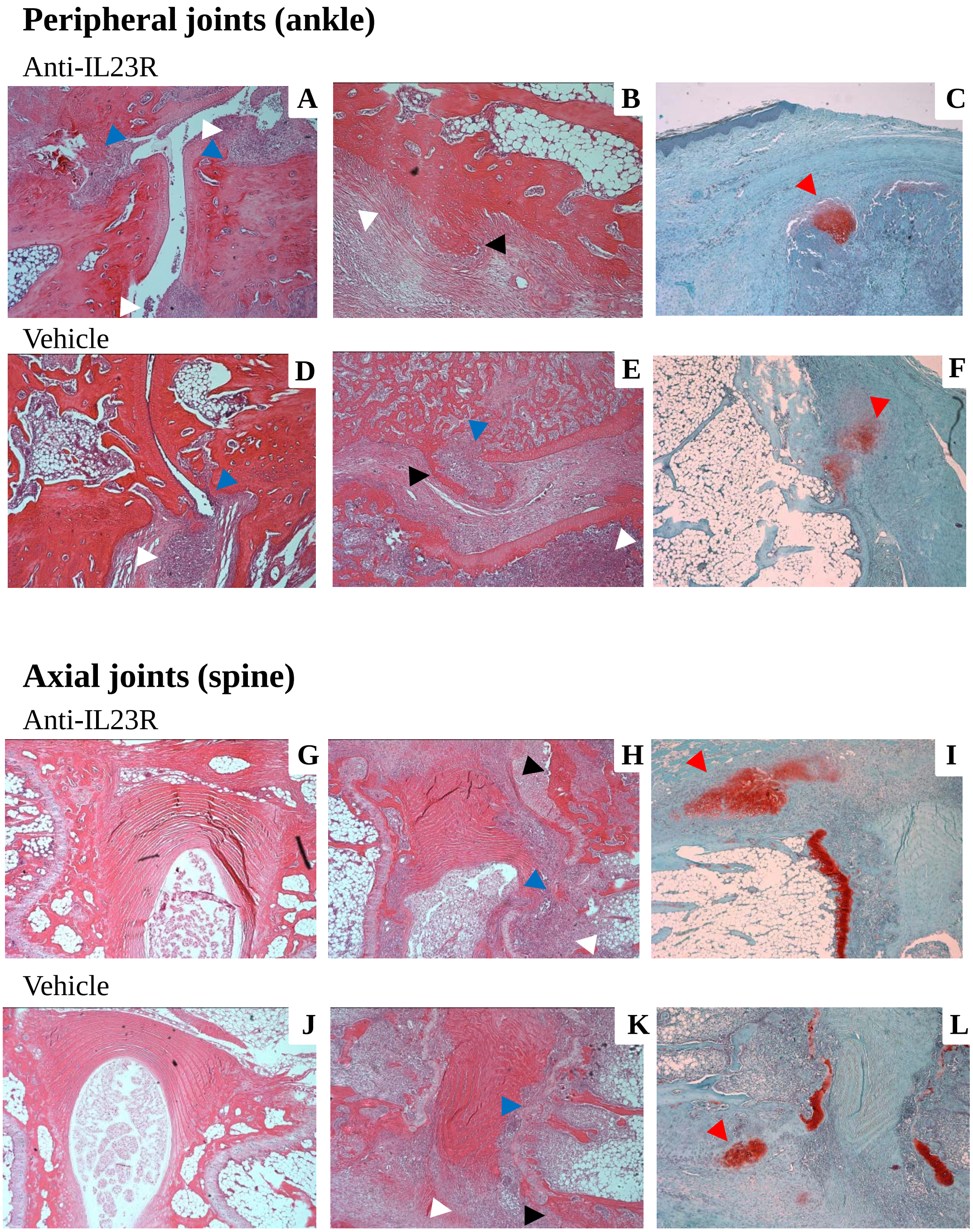

Suppl. Fig. 2 Histological pictures of HE staining and Safranin O staining on peripheral and axial joints after therapeutic treatment. Both in anti-IL23R treated peripheral and axial joints (A-C and G-I) as well as vehicle treated peripheral and axial joints (D-F and J-L) we find pathological processes including inflammation (white arrowhead), destruction (blue arrowhead), new bone formation (black arrowhead) and the presence of hypertrophic chondrocytes (red arrowhead). In case of the axial joints, not all vertebrae are affected (G and J).
