# Supplementary material for: The Initiation, but Not the Persistence, of Experimental Spondyloarthritis Is Dependent on Interleukin-23 Signaling
Source: Front Immunol. 2018 Jul 9;9:1550. doi: 10.3389/fimmu.2018.01550 (PMC6046377; doi:10.3389/fimmu.2018.01550)
Supplement: Supplementary file 3 [file image_3.pdf]

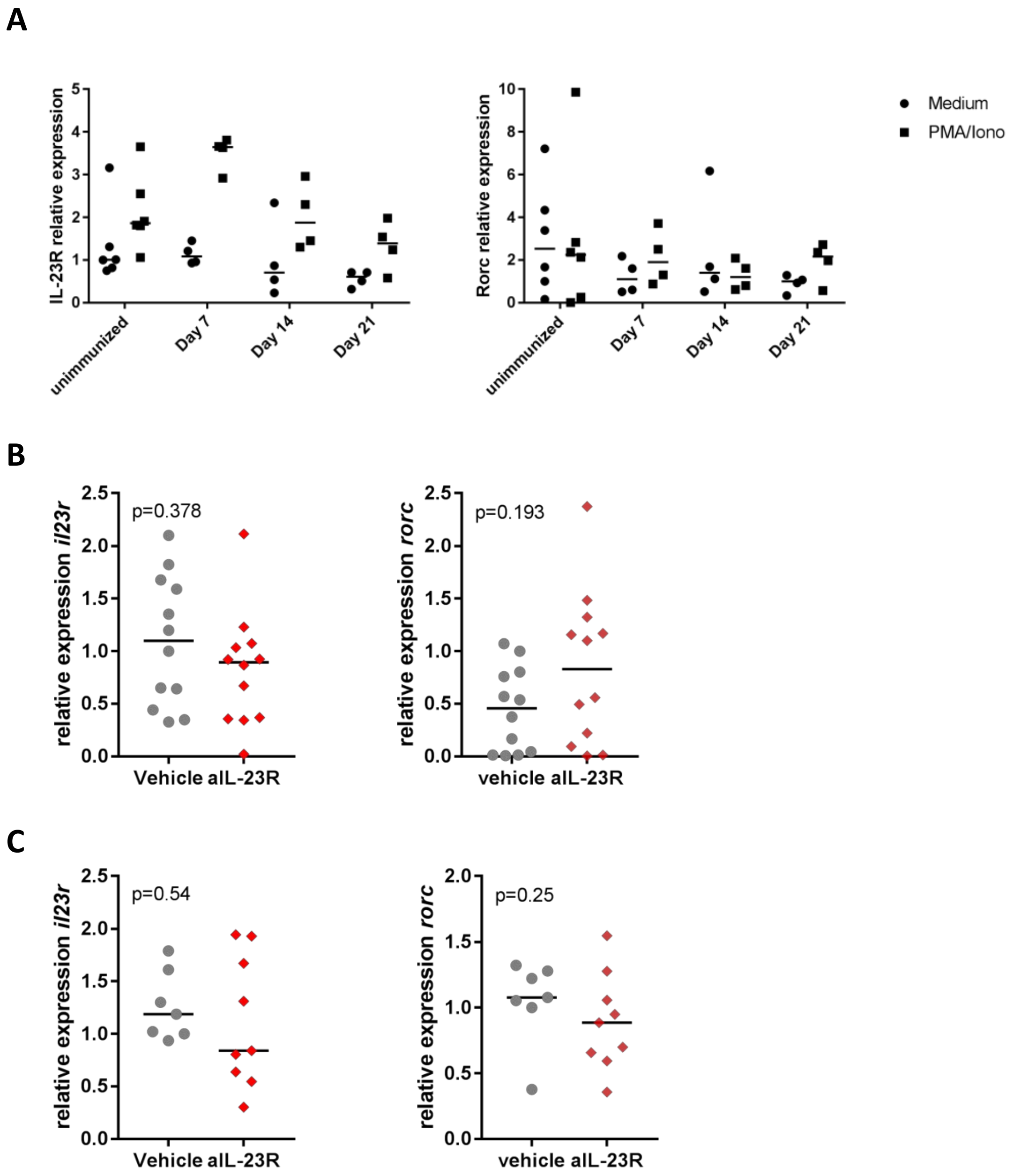

Suppl. Fig. 3 IL-23R and RorC gene expression. A. IL-23R and Rorc gene expression measured by qPCR in draining inguinal lymph nodes after immunization, but before disease onset. Cells were used unstimulated or after 6 hour stimulation with 10 ng/ml PMA and 1 µg/ml ionomycin. Each dot represents a rat, n=4-6/group. **B.** Gene expression of specifically IL-23R and RorC in popliteal lymph nodes in the prophylactic study. Each dot represents 1 rat (n=12/group) **C.** Gene expression of specifically IL-23R and RorC in popliteal lymph nodes in the therapeutic study. Each dot represents 1 rat (n=7-10/group)
