# Supplementary material for: The Initiation, but Not the Persistence, of Experimental Spondyloarthritis Is Dependent on Interleukin-23 Signaling
Source: Front Immunol. 2018 Jul 9;9:1550. doi: 10.3389/fimmu.2018.01550 (PMC6046377; doi:10.3389/fimmu.2018.01550)
Supplement: Supplementary file 4 [file image_4.pdf]

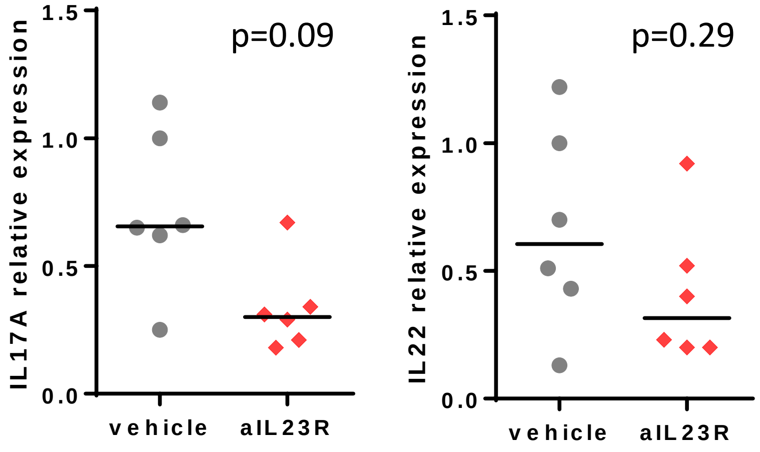

Suppl. Fig. 4 Gene expression analysis splenocytes after prophylactic treatment. IL-17A and IL-22 expression upon treatment in splenocytes (aIL23R vs vehicle).
