# Supplementary material for: The Initiation, but Not the Persistence, of Experimental Spondyloarthritis Is Dependent on Interleukin-23 Signaling
Source: Front Immunol. 2018 Jul 9;9:1550. doi: 10.3389/fimmu.2018.01550 (PMC6046377; doi:10.3389/fimmu.2018.01550)
Supplement: Supplementary file 5 [file image_5.pdf]

A

gene	fold down	p-value
Mmp3	-6.42	ns
Ccl20	-5.45	0.0145
Il17a	-4.78	ns
Il22	-4.36	0.0254
Socs3	-2.24	0.0226
Ifng	-2.12	0.0154
Il23r	-1.89	ns
Il4	-1.72	ns
Clec7a	-1.70	ns
Il17re	-1.65	ns

gene	fold down	p-value
Il12rb1	-1.63	ns
Icos	-1.59	ns
Rorc	-1.59	ns
Il6	-1.55	ns
Il2	-1.47	ns
Il21	-1.45	ns
Ccr4	-1.43	ns
Socs1	-1.39	ns
Isg20	-1.31	ns
Ccl7	-1.29	ns

gene	fold down	p-value
Csf2	-1.27	ns
Cd2	-1.23	ns
Il7r	-1.23	ns
Ccl2	-1.19	ns
Il18	-1.18	ns
Jak2	-1.17	ns
Il1b	-1.15	ns
Foxp3	-1.14	ns
Rora	-1.14	ns
Il1r1	-1.13	ns

gene	fold down	p-value
Icam1	-1.11	ns
Il17ra	-1.11	ns
Stat3	-1.10	ns
Mmp9	-1.06	ns
Tbx21	-1.06	ns
Il12rb2	-1.05	ns
Il17rc	-1.04	ns
Myd88	-1.04	ns
Tnf	-1.03	ns
Il12b	-1.02	ns

gene	fold down	p-value
Cd8a	-1.01	ns
Stat5a	-1.00	ns

B

gene	fold up	p-value
Il9	3.91	ns
Il5	2.45	0.0324
Il13	2.44	ns
Cxcl2	2.09	0.0112
Il3	1.54	ns
Cd40lg	1.52	ns
Il17c	1.48	ns
Il25	1.47	ns
Ccl22	1.44	ns
Il10	1.44	ns

gene	fold up	p-value
Il17f	1.41	ns
Il17rb	1.37	ns
Syk	1.37	ns
Il23a	1.30	ns
Il17b	1.29	ns
S1pr1	1.28	ns
Cd28	1.27	ns
Ccl1	1.26	ns
Tlr4	1.24	ns
Csf3	1.21	ns

gene	fold up	p-value
Cxcl12	1.21	ns
Ccr6	1.20	ns
Cd4	1.20	ns
Cxcl1	1.20	ns
Nfkb1	1.20	ns
Stat6	1.20	ns
Cd34	1.19	ns
Nfatc2	1.09	ns
Tgfb1	1.09	ns
Jak1	1.08	ns

gene	fold up	p-value
Gata3	1.07	ns
Cxcl6	1.06	ns
Il27	1.06	ns
Runx1	1.06	ns
Cebpb	1.05	ns
Traf6	1.05	ns
Ccr2	1.04	ns
Cx3cl1	1.04	ns
Irf4	1.04	ns
Il15	1.03	ns

gene	fold up	p-value
Il6r	1.02	ns
Stat4	1.01	ns

Suppl. Fig. 5 Gene expression analysis popliteal lymph nodes after prophylactic treatment (aIL23R vs vehicle). A. Genes that were down regulated upon aIL-23R treatment, measured by qPCR arrays in popliteal lymph nodes (n=6/group). **B.** Genes that were up regulated upon aIL-23R treatment, measured by qPCR arrays in popliteal lymph nodes (n=6/group).
