# Supplementary material for: The Initiation, but Not the Persistence, of Experimental Spondyloarthritis Is Dependent on Interleukin-23 Signaling
Source: Front Immunol. 2018 Jul 9;9:1550. doi: 10.3389/fimmu.2018.01550 (PMC6046377; doi:10.3389/fimmu.2018.01550)
Supplement: Supplementary file 6 [file image_6.pdf]

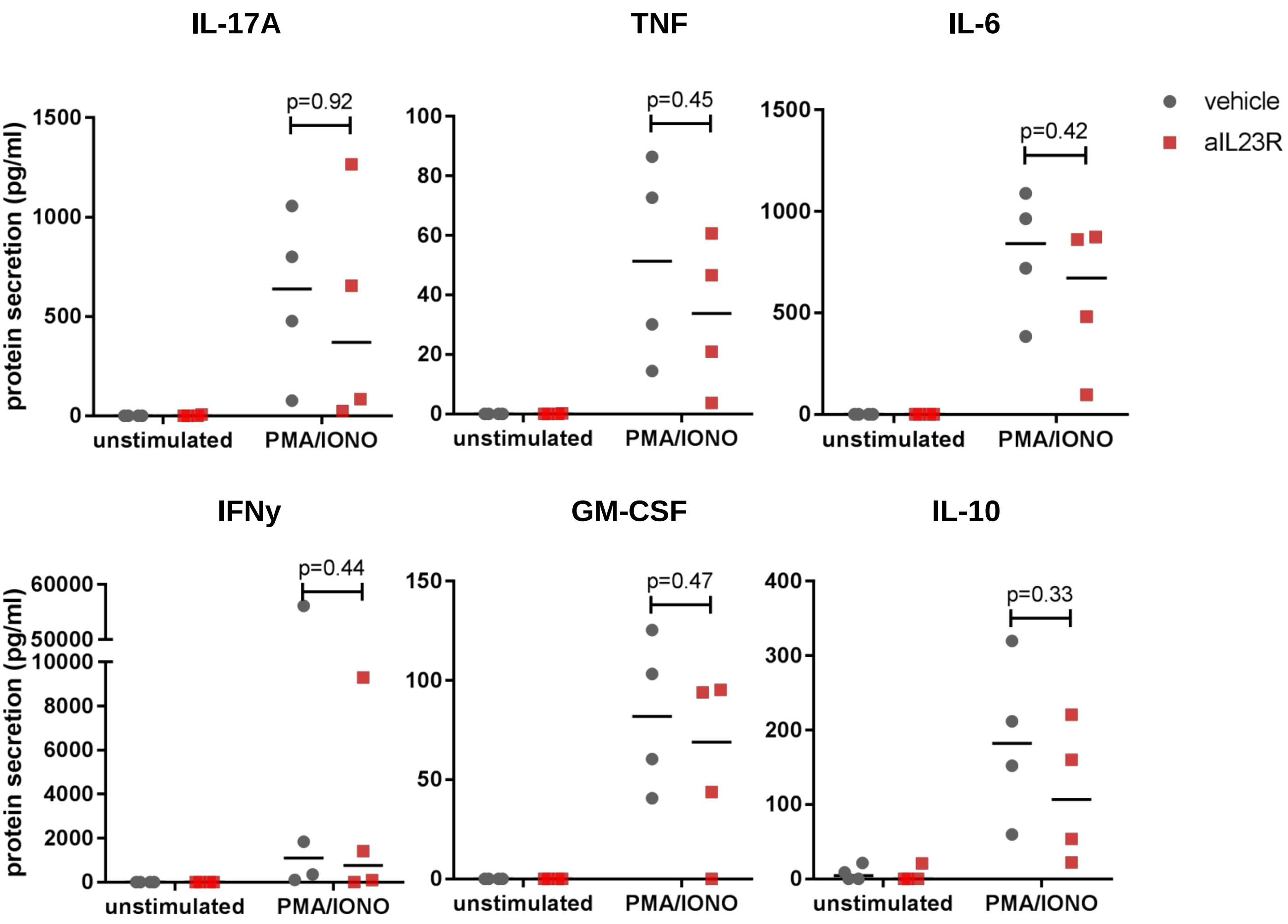


**Suppl. Fig. 6 Cytokine expression measured in supernatant from ex vivo restimulated draining lymph node cells (Luminex data).** Popliteal lymph node cells were restimulated with 10 ng/ml PMA and 1 µg/ml ionomycin for 48 hours or kept in medium alone (unstimulated).
