# Supplementary material for: The Initiation, but Not the Persistence, of Experimental Spondyloarthritis Is Dependent on Interleukin-23 Signaling
Source: Front Immunol. 2018 Jul 9;9:1550. doi: 10.3389/fimmu.2018.01550 (PMC6046377; doi:10.3389/fimmu.2018.01550)
Supplement: Supplementary file 7 [file image_7.pdf]

A

gene	fold down	p-value
Il17a	-1.66	ns
Ifng	-1.65	ns
Csf2	-1.60	ns
Ccl20	-1.59	ns
Il23r	-1.56	ns
Cxcl6	-1.40	ns
Il9	-1.36	ns
Clec7a	-1.35	ns
Il4	-1.33	ns
Rorc	-1.30	ns

gene	fold down	p-value
Il12rb1	-1.28	ns
Il17re	-1.28	ns
Rora	-1.25	ns
Mmp3	-1.23	ns
Ccl1	-1.20	ns
Il18	-1.19	ns
Il21	-1.19	ns
Il12rb2	-1.18	ns
Tbx21	-1.18	ns
Ccr2	-1.15	ns

gene	fold down	p-value
Cebpb	-1.15	ns
Il17rc	-1.15	ns
Il17ra	-1.13	ns
Icos	-1.12	ns
Mmp9	-1.12	ns
Nfatc2	-1.12	ns
Il25	-1.11	ns
Ccr6	-1.10	ns
Cd34	-1.10	ns
Cxcl12	-1.10	ns

gene	fold down	p-value
Il1b	-1.09	ns
Jak2	-1.09	ns
Stat4	-1.09	ns
Syk	-1.09	ns
Ccr4	-1.07	ns
Icam1	-1.07	ns
Il1r1	-1.07	ns
Il6	-1.07	ns
Cx3cl1	-1.06	ns
Il12b	-1.06	ns

gene	fold down	p-value
Il6r	-1.06	ns
Il17c	-1.05	ns
Il2	-1.05	ns
Il22	-1.04	ns
Il3	-1.04	ns
Stat6	-1.03	ns
Tnf	-1.03	ns
Ccl7	-1.02	ns
Il17rb	-1.02	ns
Il7r	-1.02	ns

gene	fold down	p-value
Stat3	-1.02	ns
Traf6	-1.02	ns
Foxp3	-1.01	ns
Il15	-1.01	ns
Nfkb1	-1.01	ns
Tgfb1	-1.01	ns

B

gene	fold up	p-value
Cd4	1.00	ns
Tlr4	1.01	ns
Stat5a	1.02	ns
Ccl2	1.03	ns
Jak1	1.03	ns
Myd88	1.03	ns
Irf4	1.05	ns
Cd2	1.07	ns
Il23a	1.08	ns
Cd8a	1.09	ns

gene	fold up	p-value
Cd28	1.10	ns
Runx1	1.11	ns
Cd40lg	1.13	ns
Socs1	1.13	ns
Il27	1.14	ns
Gata3	1.17	ns
S1pr1	1.18	ns
Socs3	1.19	ns
Isg20	1.21	ns
Ccl22	1.23	ns

gene	fold up	p-value
Csf3	1.36	ns
Cxcl1	1.37	ns
Il10	1.44	ns
Cxcl2	1.53	ns
Il5	1.62	ns
Il17b	1.72	ns
Il13	2.17	ns
Il17f	2.22	ns

Suppl. Fig. 7 Gene expression analysis popliteal lymph nodes after therapeutic treatment (aIL23R vs vehicle). A. Genes that were down regulated upon aIL-23R treatment, measured by qPCR arrays in popliteal lymph nodes (n=4-5/group). **B.** Genes that were up regulated upon aIL-23R treatment, measured by qPCR arrays in popliteal lymph nodes (n=4-5/group).
